# Immune Cluster and PPI Network Analyses Identified CXCR3 as a Key Node of Immunoregulation in Head and Neck Cancer

**DOI:** 10.3389/fonc.2020.564306

**Published:** 2021-01-15

**Authors:** Ping Wang, Yanli Wang, Yuanjun Jiang, Minghong Li, Guang Li, Qiao Qiao

**Affiliations:** ^1^ Department of Radiation Oncology, The First Hospital of China Medical University, Shenyang, China; ^2^ Department of Urology, The First Hospital of China Medical University, Shenyang, China; ^3^ Department of Otolaryngology, The First Hospital of China Medical University, Shenyang, China

**Keywords:** head and neck cancer, tumor microenvironment, single-sample gene set enrichment analysis, the Cancer Genome Atlas, prognosis, treatment

## Abstract

The tumor microenvironment (TME) is significantly associated with clinical outcomes and therapeutic efficacy. However, the landscape of the head and neck cancer (HNC) microenvironment is not fully understood. Therefore, we divided HNCs into three classes according to differences in the TME to determine effective personalized treatments. We explored the immune landscape of head and neck cancer by analysing the gene expression profile of 501 cases from the Cancer Genome Atlas (TCGA) data portal and validated our findings in 270 cases from the Gene Expression Omnibus (GEO) database. The levels of immune components in the tumor microenvironment were evaluated *via* single-sample gene set enrichment (ssGSEA) analysis. The HNCs were clustered into an Immunity-H group, Immunity-M group and Immunity-L group according to 40 immune components in the tumor microenvironment. DNA damage and HLA genes play an important role in immune regulation. The patients in the Immunity-H group had a favourable survival compared with patients in the Immunity-M group and the Immunity-L group. The patients in the Immunity-H group and Immunity-M group could benefit from radiotherapy. In addition, the Immunity-L group showed the lowest immunophenoscore and had poor response to anti-PD-1 treatment. CXCR3 was demonstrated to be downregulated in the Immunity-L group, which was related to shorter OS in the TCGA and GEO databases, suggesting CXCR3 as a potential therapeutic target. Taken together, our findings proposed three new microenvironment-related phenotypes of HNCs and suggested that CXCR3 played a major role in immune regulation and could be a novel therapeutic target, providing a reference for clinical decisions and research directions in the future.

## Introduction

Head and neck squamous cancer (HNSCC) ranks seventh in global cancer incidence, and 890,000 people were diagnosed with HNSCC in 2018 (1). On the basis of traditional treatments, including surgical resection and chemoradiation, the 5-year survival rate of HNC remains 40%–50% (2). Other than cetuximab, a monoclonal antibody (mAb) targeting epidermal growth factor receptor (EGFR), no other new targeted therapies have been approved for HNSCC for decades. Cetuximab monotherapy efficiency is only 10%~15%, and there are no known biomarkers for predicting response (3, 4). At present, immunotherapy has been approved by the FDA and EMEA to treat recurrent and metastatic patients with HNSCC (5). However, regardless of the type of treatment, some people do not benefit or experience associated side effects. Therefore, it is crucial to explore new therapeutic targets and identify therapeutic methods that are suitable for specific groups of people.

The tumor microenvironment (TME) plays an important role in cancer growth, metastasis and response to therapy. The tumor microenvironment is composed of cancer cells, immune cells, stromal tissues, the extracellular matrix and other components (6). Chemokine and cytokine signalling in the TME regulates tumor behaviour and response to therapy, affecting the interactions among immune cells (7). Immune cells can change their status according to different tumor microenvironments. Sometimes, immune cells fail to clear tumor cells due to the immunosuppressive status of the tumor microenvironment (8).

In our study, we aimed to apply the ssGSEA method to evaluate the levels of various immune components, including immune cells, factors and pathways, in the tumor microenvironment and identify people with different immune statuses through k-means clustering to explore the optimal treatment plans in HNSCC.

## Materials and Methods

### Data Collection and Collation

We collected the gene expression data and clinical data of the TCGA-HNSC cohort from https://genome-cancer.ucsc.edu/. The CNV data and mutation data of these patients with head and neck squamous cancer were downloaded from the publicly available TCGA database *via* the GDC Data Portal. We ultimately analyzed 501 TCGA-HNSC patients after excluding patients who did not have survival data.

### Single-Sample Gene Set Enrichment Analysis (ssGSEA) and Clustering

We obtained marker gene sets for 40 immune components, including immune cells, immune-related factors and immune pathways, in the tumor environment from a previously published study (9) and the ImmPort database (10). We calculated the enrichment scores of 40 immune-related signatures in 501 patients by using the R package “gsva” (11). According to different immune scores, we used k-means clustering to divide these patients into three groups: the Immunity-high group, the Immunity-moderate group and the Immunity-low group. The immune scores, stromal scores and tumor purity were calculated based on the ESTIMATE algorithm (12). The TIMER database (https://cistrome.shinyapps.io/timer/) was used to calculate the infiltration levels of six immune cells, including CD4^+^ T cells, CD8^+^ T cells, B cells, macrophages, neutrophils, and dendritic cells(DCs) according to different gene sets (13, 14).

### Calculation of DNA Damage and Immunogenomic Indicators

Aneuploidy, HRD (homologous recombination deficiency), CNA burden (copy number variation burden), ITH (intratumor heterogeneity), and SNV-related neoantigen data were obtained from a published study (15). The tumor mutational burden (TMB) was calculated as the number of nonsynonymous protein-coding variants divided by the total sequenced genome length according to the mutation data (16).

### Response to Therapy

The patients were divided into two groups *via* k-means clustering based on the expression level of 31 genes associated with radiosensitivity (17). A radioresistant group (RR group) and a radiosensitive group (RS group) were identified based on prognosis under radiotherapy. The immunophenoscore (IPS) (18) and chemosensitivity (19) were determined from previously published studies.

### DEG Screening, PPI Network Construction, and Hub Gene Identification

DEGs between the Immunity-H group and Immunity-L group were identified with the “edgeR” package (20) and the criterion |logFC| > 1 and P < 0.05. A web tool, the STRING database (https://string-db.org/), was used to build a protein-protein interaction network for the DEGs (21), and the minimum required interaction score was set as 0.400. The top 10 hub genes were identified by the “cytohubba” tool in Cytoscape software 3.6.1.5 (22, 23).

### Gene Ontology and KEGG Pathway Enrichment Analyses

The associated molecular functions (MFs), biological processes (BPs), cellular components (CCs) and pathways for the top 10 hub genes were annotated by the R packages “clusterProfiler” and “org.Hs.eg.db” (24). A false discovery rate (FDR) value <0.05 was set as the enrichment cut-offs to screen for meaningful enrichment results. The enrichment results were visualized *via* the R package “ggplot2R”. Survival analysis of the top 10 hub genes was performed using the R software package “survival”.

### Immunohistochemistry

A total of 53 biopsy samples from patients with primary head and neck cancer were collected at the First Hospital of China Medical University between 2005 and 2011. Using 4 μm-thick sections, immunohistochemistry was performed. The antibody used was rabbit anti-human CXCR3 polyclonal antibody (BA0759, 1:300 dilution; WB-BIO, Wuhan, China). The staining intensity and percentage of positive cells was estimated. The proportion of positive cells greater than 5% is considered positive based on previous studies (25).

### Correlation Analysis

We evaluated the correlation between the expression of CXCR3 and other hub genes in HNSCC through GEPIA (http://gepia.cancer-pku.cn/index.html) based on the TCGA database (26). Pearson’s correlation coefficient analysis was used to define correlations.

### CNV Analysis

The copy number variation score processed by GISTIC2.0 was downloaded from the TCGA database *via* the GDC Data Portal. Segment values larger than 0.3 were defined as “1”, and those less than -0.3 were defined as “-1”; segment values between -0.3 and 0.3 were defined as “0”. The relationship between gene copy number variation and immune infiltration was analyzed with TIMER (27).

### GEO Validation Dataset

Validation data (GSE65858 and GSE41613) were downloaded from the Gene Expression Omnibus database (www.ncbi.nlm.nih.gov/geo). The GSE65858 data were analyzed to validate the immune classes. The GSE41613 data were analyzed to validate the prognostic value of CXCR3.

### Statistical Analysis

The chi-square test was adopted to identify the significantly different copy number variants (CNVs) between the Immunity-H group and the Immunity-L group. An independent samples t-test was employed to compare two groups, while one-way ANOVA test was employed to compare multiple groups. All analyses were performed with R software (Version 3.6.1) and IBM SPSS Statistics software (Version 26). P-values <0.05 were considered statistically significant.

## Results

### Identification and Validation of the Immune Class Clustering by 40 Immune Components in the HNSCC Environment

**Figure 1:**
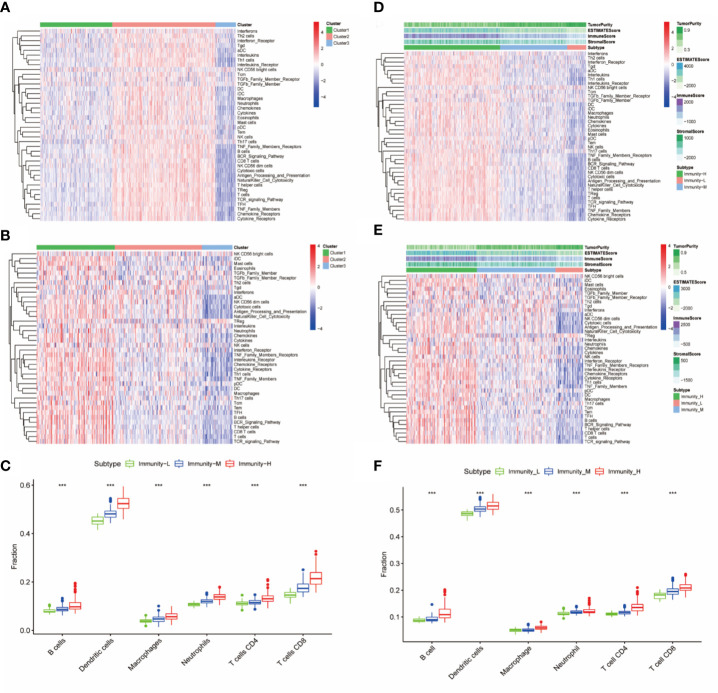
Identification and validation of immune classes of head and neck squamous cancer (HNSCC) in the Cancer Genome Atlas (TCGA) and Gene Expression Omnibus (GEO) cohorts. **(A)** K-means clustering of HNSCC samples in the TCGA cohort based on the scores of 40 immune components calculated by ssGSEA. **(B)** Differences in tumor purity, ESTIMATE scores, immune scores and stromal scores among the three immune classes. **(C)** Differences in the levels of six immune cells (B cells, CD4+ T cells, CD8+ T cells, neutrophils, macrophages, and dendritic cells) among the three immune classes. **(D)** K-means clustering of HNSCC samples in the GSE65858 cohort based on the scores of 40 immune components calculated by ssGSEA. **(E)** Differences in tumor purity, ESTIMATE scores, immune scores and stromal scores among the three immune classes in the GSE65858 cohort. **(F)** Differences in the levels of six immune cells (B cells, CD4+ T cells, CD8+ T cells, neutrophils, macrophages, and dendritic cells) among the three immune classes. The p-value indicates the different degrees among the three clusters. ***p < 0.001.

Immune cells, immune factors and immune pathways participate in the immune response together to prevent the occurrence and progression of tumors in the immune microenvironment of HNSCC. We evaluated the levels of 40 immune components, including immune cells, immune factors, and immune pathways, by the ssGSEA method according to the transcriptomes of 501 TCGA-HNSC patients. These patients were clustered into three clusters (Immunity-H: 264; Immunity-M: 185; Immunity-L: 52) based on the 40 immune components by k-means clustering (**Figure 1A**). To validate the reliability of the immune classes, we first compared the immune scores, stromal scores and tumor purity among the three immunity classes. The Immunity-H group showed the highest immune scores and stromal scores and the lowest tumor purity; in addition, the Immunity-L group showed the lowest immune scores and stromal scores and the highest tumor purity (**Figure 1B**). The distributions of six immune cells calculated in the TIMER database were in agreement with our immune classes (**Figure 1C**). We further validated our expression profile-based clustering by the same approach in 270 samples from GEO datasets (**Figure 1D**). The immune scores, stromal scores, and tumor purity and the distributions of six immune cells were consistent with the immune clustering (**Figures 1E, F**). Overall, we demonstrated that the HNSCC samples had three different immune statuses when assessed by 40 immune components.

### HLA Expression and DNA Damage Were Associated With Immune Class

**Figure 2:**
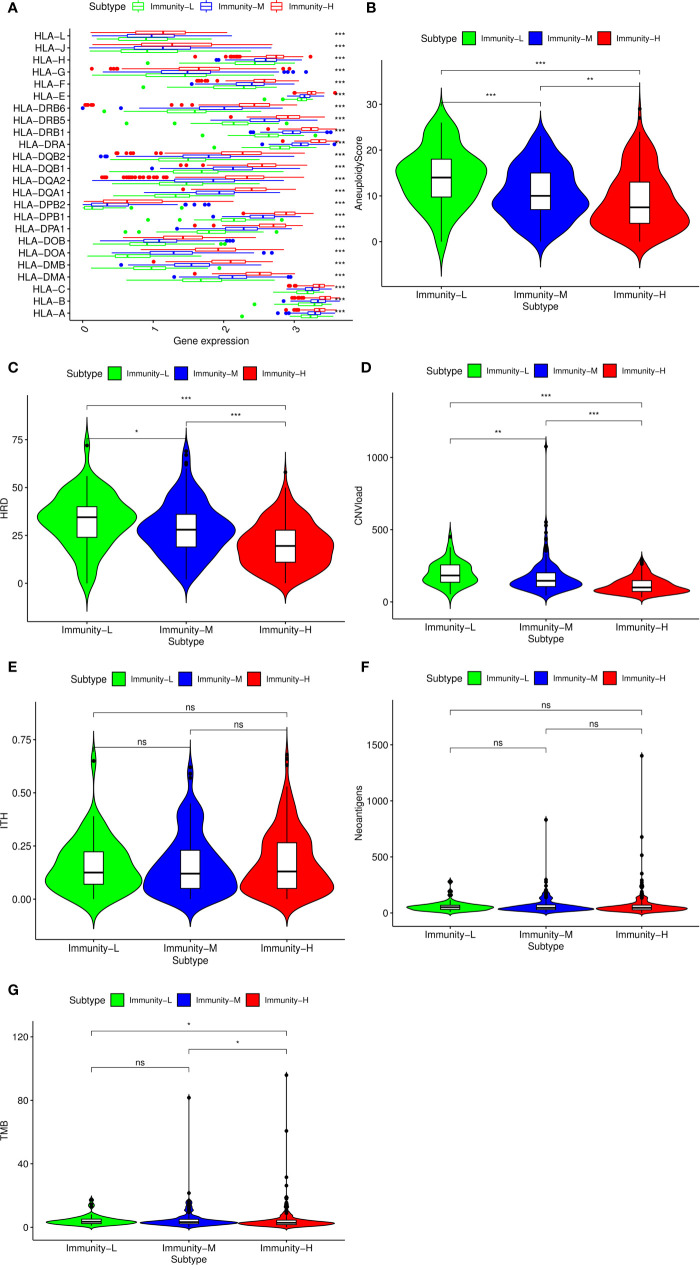
Potential immunoregulatory mechanisms of head and neck squamous cancer (HNSCC). **(A)** Correlation between HLA gene expression and immune class. Comparison of aneuploidy score **(B)**, homologous recombination deficiency (HRD) score **(C)**, CNV burden **(D)**, intratumor heterogeneity (ITH) score **(E)**, SNV neoantigen burden **(F)**, and tumor mutational burden (TMB) **(G)** among the three classes. The p-value indicates the different degrees among the three clusters.***p < 0.001; **p < 0.01; *p < 0.05; ns, p > 0.05.

Considering previous publications (15, 28), there are at least three aspects associated with immune status: HLA gene expression, DNA damage and immunogenicity. HLA (human leukocyte antigen) on the surface of many immune cells plays an important role in activating cellular and humoral immunity (29). We compared HLA gene expression among the three classes and found that all HLA genes were expressed at a high level in the Immunity-H group and at a low level in the Immunity-L group (P<0.001) (**Figure 2A**), validating that the absence of the HLA gene contributed to the low immune status. In addition to HLA gene expression, DNA damage and immunogenicity are also associated with the immune response (15). To estimate whether DNA damage and immunogenicity affected the immune status of the HNSCC samples, we investigated the correlation between the immune class and these factors. DNA damage can be considered in 4 major categories: aneuploidy, homologous recombination deficiency, copy number variation burden and intratumor heterogeneity. Low immune status was significantly associated with high aneuploidy score, high HRD and high CNA burden but had no relevance to ITH (**Figures 2B–E**). For immunogenicity, the differences in the tumor mutational burden and SNV-related neoantigen burden were not as large as those in homologous recombination deficiency and copy number variation burden (**Figures 2F, G**). In general, HLA expression and DNA damage were significantly associated with the immune class.

### Prognostic Value of 40 Immune Components in HNSCC

**Figure 3:**
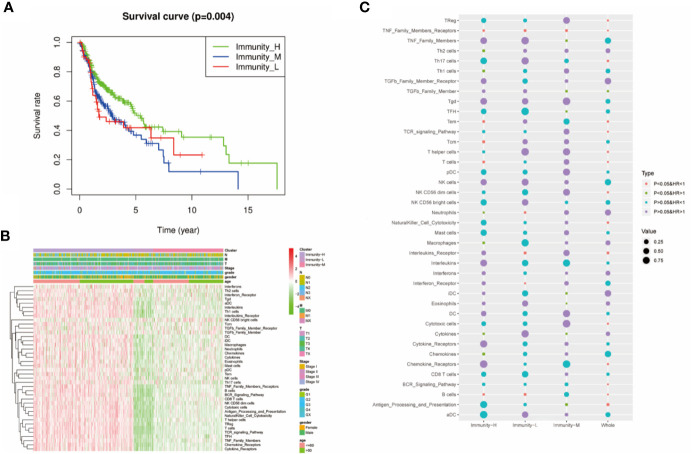
Prognostic significance of 40 immune components in head and neck squamous cancer (HNSCC). **(A)** Kaplan–Meier curves of OS among the three immune clusters. **(B)** Heatmap showing the relationship of immune clusters classed by 40 immune components and the clinical factors including age, gender, TNM stage, and grade. There is no obvious correlation between the immune classes and clinical factors. **(B)** GO analysis based on the significant genes in the comparison between low- and high-risk groups. **(C)** Univariate Cox analysis revealing the prognostic value of each immune component for OS in all cohorts and each immune class. The color represents the different HR and P value.

We performed survival analysis among the three groups by different methods, and the results demonstrated that the immune classes were associated with prognosis. Kaplan–Meier analysis showed that the Immunity-H group had better survival than the Immunity-M group and Immunity-L group (P<0.01) (**Figure 3A**). The multivariate Cox proportional hazards model also revealed that the Immunity-M group and Immunity-L group independently predicted worse overall survival in HNSCC than the Immunity-H group (**Table 1**). In addition, we investigated the association between clinical factors and immune class. There were similar clinicopathologic characteristics among the three immune classes (**Figure 3B**). Considering that the immune microenvironment is dynamic, we compared the prognostic value of 40 immune components in the three different immune statuses (**Figure 3C**). In the whole HNSCC cohort, most immune components were related with survival. However, 40 immune components showed different prognostic effects with different immune statuses, for example, the Th1 cells, Th2 cells, and macrophages were associated with prognosis only in the Immunity-H group. Moreover, neutrophils predicted a worse prognosis in the Immunity-H group and a better prognosis in the Immunity-L group and are thus worth studying further.

**Table 1:** Univariate and multivariate cox proportional hazards model for OS.

Univariate and multivariate cox proportional hazards model for OS					
Variables		Univariate		Multivariate	
		HR (95%CI)	P	HR (95%CI)	P
Ages(years)		1.02(1.008–1.033)	**0.002**	1.121(1.008–1.033)	**0.001**
Clinical stage	Stage I	Ref			
	Stage II	1.12(0.5–2.507)	0.783		
	Stage III	1.318(0.594–2.926)	0.497		
	Stage IV	1.380(0.644–2.958)	0.408		
Histologic grade	G1	Ref			
	G2	1.669(1.063–2.620	**0.026**		
	G3	1.431(0.876–2.337)	0.152		
	G4	0.930(0.378–2.289)	0.875		
Cluster	Immunity-H	Ref		Ref	
	Immunity-M	1.554(1.168–2.068)	**0.003**	1.543(1.16–2.053)	**0.003**
	Immunity-L	1.588(1.037–2.434	0.034	1.658(1.081–2.542)	**0.021**

The p value was bold when it <0.05.

### Therapeutic Strategies According to Immune Class

**Figure 4:**
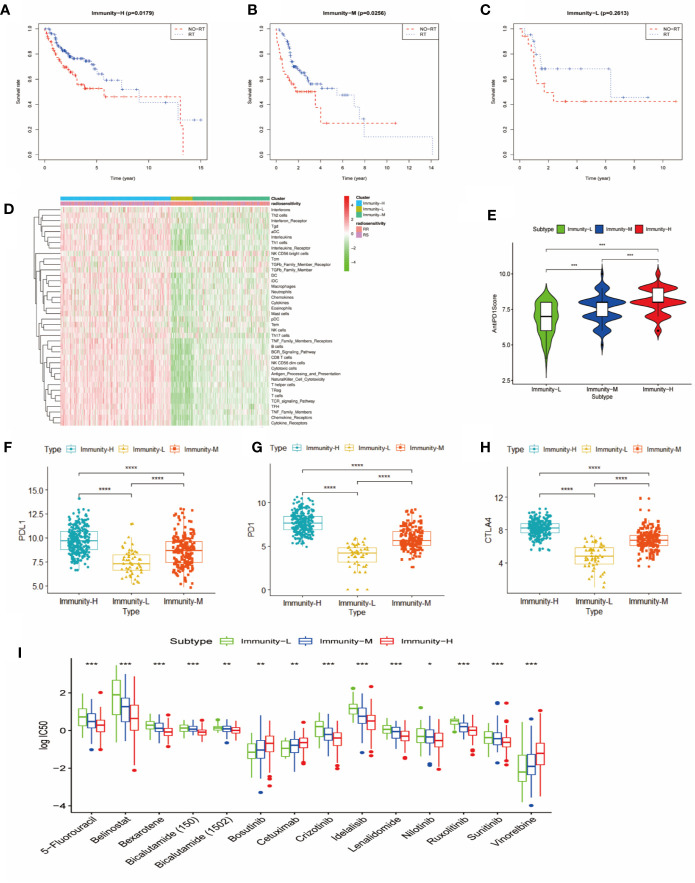
Comparison of therapy response among the immune classes. **(A)** Kaplan-Meier curves for OS of head and neck squamous cancer (HNSCC) patients in the Immunity-H group. Those patients treated with radiotherapy had better survival than patients without radiotherapy. **(B)** Kaplan-Meier curves for OS of HNSCC patients in the Immunity-M group. Those patients treated with radiotherapy had better survival than patients without radiotherapy. **(C)** Kaplan-Meier curves for OS of HNSCC in the Immunity-L group. There was no significant difference in OS between patients treated with radiotherapy and without radiotherapy. **(D)** Distribution of radiosensitivity (RS) and radioresistance (RR) patients in the three immune classes. A comparison of anti-PD-1 immunophenoscore **(E)**, PD-L1 expression **(F)**, PD1 expression **(G)**, and CTLA4 expression **(H)** between the three immune classes is shown. **(I)** Chemosensitivity according to three different immune classes ****p < 0.0001; ***p < 0.001; **p < 0.01; *p < 0.05.

We further explored the therapeutic effects of common treatments in HNSCC. In the Immunity-H group and the Immunity-M group, patients treated with radiotherapy exhibited better overall survival rates than patients without radiotherapy (p<0.05) (**Figures 4A, B**), whereas there were no significant differences in the Immunity-L group (p=0.2613) (**Figure 4C**). In addition, the proportion of radiosensitive patients in the Immunity-H group and the Immunity-M group was obviously greater than that in the Immunity-L group (**Figure 4D**). Charoentong et al (18). proposed an IPS for defining patients likely to respond to anti-PD-1 therapy. As illustrated in **Figure 4E**, the IPS score decreased as the immune status decreased (p<0.001); in addition, the immunotherapy markers PD-L1, PD1, and CTLA-4 also decreased as the immune status decreased (p<0.0001) (**Figures 4F–H**). We next estimated the chemosensitivity in subgroups, and the log-transformed IC50 values are shown in **Figure 4I**. Most drugs were most effective in the Immunity-H group, including 5-fluorouracil, belinostat, bexarotene, bicalutamide, idelalisib, lenalidomide, nilotinib, and ruxolitinib. Only cetuximab, bosutinib, and vinorelbine were effective in the Immunity-L group. As a result, the low immune status predicted worse efficacy of administered treatments than the moderate and high immune statuses.

### PPI Network Construction Revealed That CXCR3, the Top Hub Gene in Immunoregulation, Was Associated With Prognosis

**Figure 5:**
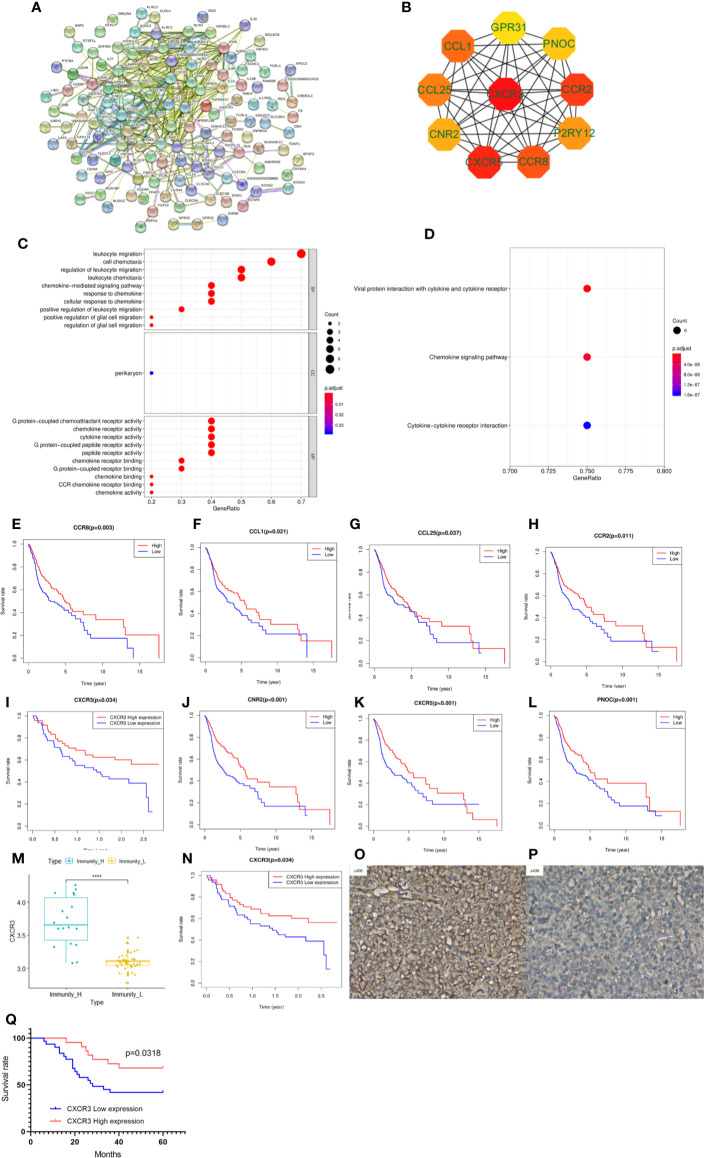
Protein-protein interaction (PPI) network, hub gene identification and prognostic value validation. **(A)** PPI network of differentially expressed genes between the Immunity-L group and the Immunity-H group. **(B)** The top ten hub genes were selected by the “cytohubba” tool in Cytoscape software, and nodes with higher degrees are displayed in bright red. **(C)** Gene ontology analysis of the 10 hub genes. Biological process terms for 10 genes. **(D)** KEGG pathway enrichment analysis of the 10 hub genes. Molecular function terms for 10 hub genes. The prognostic value of CCR8 **(E)**, CCL1 **(F)**, CCL25 **(G)**, CCR2 **(H)**, CXCR3 **(I)**, CNR2 **(J)**, CXCR5 **(K)**, and PNOC **(L)** is shown. **(M)** CXCR3 was expressed at lower levels in the Immunity-L group than in the Immunity-H group in the GSE41613 validation cohort. **(N)** Low expression of CXCR3 predicted worse prognosis than high expression of CXCR3 in the GSE41613 validation cohort. **(O)** High CXCR3 protein expression in head and neck squamous cancer (HNSCC). **(P)** low CXCR3 protein expression in HNSCC. **(Q)** Patients with high CXCR3 expression in head and neck cancer had longer S(p < 0.05).

To explore potential therapeutic targets to reverse the immune status in the Immunity-L group, we identified 677 downregulated and 21 upregulated genes in the Immunity-L group compared with the Immunity-H group. As shown in **Figure 5A**, we constructed a PPI network composed of 177 nodes and 437 edges (**Figure 5A**). In addition, we identified the top 10 hub genes, including CXCR3, CXCR5, CCR2, CCR8, CCL1, CCL25, P2RY12, CNR2, PNOC, and GPR31 (**Figure 5B**). In terms of biological processes, these hub genes were significantly enriched in leukocyte migration and cell chemotaxis. In terms of molecular functions, these hub genes were enriched in G protein-coupled chemoattractant receptor activity, chemokine receptor activity, cytokine receptor activity, etc. (**Figure 5C**). KEGG pathway enrichment analysis showed that the hub genes were associated with viral protein interactions with cytokine and cytokine receptors, chemokine signalling pathways and cytokine-cytokine receptor interactions (**Figure 5D**).

We further analyzed the prognostic value of the 10 hub genes. Low expression of CCR8, CCL1, CCL25, CCR2, CXCR3, CNR2, CXCR5 and PNOC was associated with worse survival (p<0.05) (**Figures 5E–L**), whereas P2RY12 and GPR31 expression was not associated with survival. Moreover, CXCR3, the top hub gene, was downregulated in the Immunity-L group compared with the Immunity-H and Immunity-M groups (**Figure 5M**), and low CXCR3 expression predicted worse prognosis than high CXCR3 expression (P<0.05) (**Figure 5N**), which were validated in the GEO datasets. The correlation analysis implied that CXCR3 expression was positively correlated with the other 9 hub genes, including CXCR5 (R=0.3, P<0.05), CCR2 (R=0.76, P<0.05), CCR8 (R=0.58, P<0.05), CCL1 (R=0.15, P<0.05), CCL25 (R=0.12, P<0.05), CNR2 (R=0.52, P<0.05), PNOC (R=0.52, P<0.05), P2RY12 (R=0.65, P<0.05), and GPR31 (R=0.69, P<0.05), which further revealed that CXCR3 plays a core role in immune regulation. For a more comprehensive study, we accessed the CXCR3 expression using immunohistochemistry and CXCR3 protein was mainly expressed in the cytoplasm of cell (**Figures 5O, P**). CXCR3 positive expression was identified in 23 cases (43.39%) and positive CXCR3 status was associated with better prognosis (**Figure 5Q**).

LASSO regression analysis of the top 10 hub genes showed that the expression levels of 4 genes, P2RY12, CXCR3, PNOC, and CCR8, could be used to predict low immune status. In addition, there were significant associations between CXCR3 (p<0.001), PNOC (p=0.026), and CXCR8 expression and low immune status (p=0.005) in the multivariate logistic regression model (**Table 2**). When using the three genes to predict the risk of patients with low immune status, the accuracy was 94.2%.

**Table 2:** Four hub genes parameters in the logistic regression models.

Gene	Regression coefficient	OR	95%CI	P value
P2RY12	-1.990	0.137	0.003–7.363	0.328
CXCR3	-5.986	0.003	0.0002–0.030	0.000002
PNOC	-3.187	0.041	0.002–0.689	0.026
CCR8	-4.217	0.015	0.001–0.275	0.005

### CXCR3, CXCR5, and CCL1 Copy Number Variants Correlated With Low Immune Status

**Figure 6:**
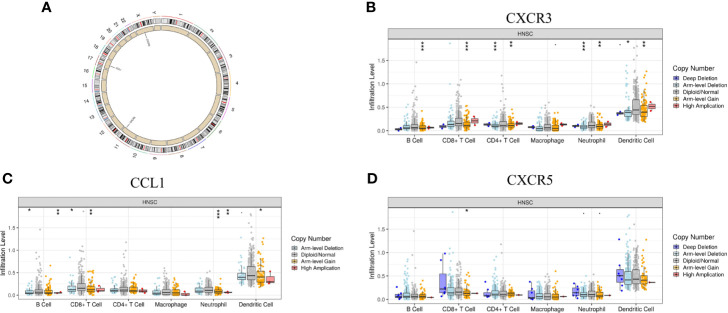
The relationship between the CNV of the 10 hub genes and immunity. **(A)** Hub genes with different CNV burdens in samples according to their chromosomal location. **(B)** CXCR3 copy number variation correlated with immune infiltration. **(C)** CCL1 copy number variation correlated with immune infiltration. **(D)** CXCR5 copy number variation correlated with immune infiltration. The p-value indicates the different degrees among the three clusters. ***p < 0.001; **p < 0.01; *p < 0.05.

In addition to transcriptome analysis, we also investigated the CNV differences of the 10 hub genes between the Immunity-L group and Immunity-H group. The CNV events related to CXCR3 on chromosome X, CCL1 on chromosome 17 and CXCR5 on chromosome in the Immunity-L group were significantly more frequent than those in the Immunity-H group (p<0.05) (**Figure 6A**). However, CNV events related to the other seven hub genes were not associated with immune class. In addition, we investigated the association between CNV events related to the three genes and immune infiltration in the TIMMER database. CXCR3 copy number variation was significantly associated with decreased levels of six immune cells (B cells, CD4 + T cells, CD8 + T cells, neutrophils, macrophages, and dendritic cells) (**Figure 6B**), and CCL1 copy number variation was significantly associated with decreased levels of 4 immune cells (B cells, CD8 + T cells, neutrophils, and dendritic cells) (**Figure 6C**), but CXCR5 copy number variation was only associated with CD8 cell level (**Figure 6D**). These results showed that CXCR3 and CCL1 copy number variations significantly regulate immunity, whereas CXCR5 copy number variations may have little relation with immunity.

## Discussion

In recent years, based on emerging evidence, solid tumors are no longer thought to exist in isolation but rather in a complicated environment called the tumor microenvironment composed of multiple cell types, including neutrophils, macrophages, regulatory T cells, and more. These cells interact with each other *via* various cytokines, chemokines, and growth factors (30). The purpose of our study was to determine the heterogeneity of HNSCC samples within different immune statuses through analysis of immune cells, factors, and pathways in the TME.

Using the scores for 40 immune components calculated by ssGSEA, we divided HNSCC samples into three immune classes, the Immunity-H group; the Immunity-M group; and the Immunity-L group, and confirmed the utility of the classifications *via* different methods and different cohorts. In previously published literature (15, 28), we found the following characteristics of the classes that might contribute to low immune status: 1, defective HLA gene expression leading to defective presentation of antigens and immune system activation; 2, DNA damage driving endogenous immune deficiency; and 3, aberrant immunogenicity. The first two reasons were confirmed in this study; nevertheless, the third one could not be confirmed in this study.

We further analyzed the prognosis of the three immune classes. The patients in the Immunity-H group had the best survival compared with patients in the Immunity-M group and the Immunity-L group. Malignant tumors promote tumor progression by suppressing effective antitumor immunity, and immune cells, factors and pathways are indispensable parts of the immune response (31). These findings could explain the different prognoses among the three immune classes. Interestingly, the 40 immune components showed prognostic effects on prognosis in different immune statuses; for example, neutrophils predicted opposite prognoses in the Immunity-H group and the Immunity-L group. Regarding this interesting phenomenon, we hypothesized that neutrophils could be differentially polarized and driven by the tumor microenvironment to transition into different subtypes with different functions in the Immunity-H group and the Immunity-L group (32). In 2019, Fridlender and colleagues (33) first suggested the description of antitumorigenic and protumorigenic neutrophils, but no definitive surface marker were identified to distinguish them. Our results further illustrated that immune cells can vary their functions to adapt to different tumor environments.

Our research also provided a guide for tailoring therapeutic strategies. First, we found that radiotherapy improved the overall survival of the patients in the Immunity-H group and the Immunity-M group, and the percentage of radiosensitive patients increased with the improvement of immune status. Similarly, most of the chemotherapy drugs were also most effective in the Immunity-H group. Recently, an increasing number of studies have indicated that immunotherapy greatly improves the prognosis of patients with advanced disease. In our research, we predicted the immunotherapy response by the anti-PD-1 score proposed by Charoentong et al. and some related markers in previous studies. Anti-PD1 treatment could revive early-stage dysfunctional T cells but not late-stage dysfunctional T cells (34), which supported our results. We found that the IPSs and immune marker levels were the lowest in the Immunity-L group compared with the Immunity-M group and the Immunity-H group, which showed that the patients in the Immunity-L group suffered from late-stage immune dysregulation and presented a low immune status because of long-term immune disorders. In general, monotherapy of radiotherapy, chemotherapy, or immunotherapy was efficacious in the Immunity-H group, and combined radiotherapy and chemotherapy or immunotherapy was suitable in the Immunity-M group; however, there were no satisfactory effects from existing treatments methods for the patients in the Immunity-L group.

To explore some potential targets to improve the prognosis of patients in the Immunity-L group, we obtained 697 DEGs *via* a comparison between the Immunity-H group and the Immunity-L group and 10 hub genes by PPI network analysis of these DEGs. The enrichment analysis of the 10 hub genes showed that the genes mainly participated in functions and pathways related to chemokines and cytokines. The 10 genes, CXCR3, CXCR5, CCR2, CCR8, CCL1, CCL25, CNR2, and PNOC, for which low expression was associated with worse survival were potential targets, and CXCR3 and CCL1 copy number variations were significantly associated with low immunity. Through validation analysis in the GEO database, we found that the chemokine receptor CXCR3 was the most valuable target. HNSCC samples with lower expression of CXCR3 had significantly shorter OS than HNSCC samples with higher expression of CXCR3, and this relation was validated in the GEO datasets. Similarly, Fangfang Chen et al. indicated that the overexpression of CXCR3 was associated with increased dendritic cell and tumor-infiltrating lymphocyte infiltration and improved OS in gastric cancer (35). However, some researchers found that higher CXCR3 expression was associated with more distant metastasis and shorter OS than lower CXCR3 expression (36, 37). Although the prognostic effects were different in different solid tumors, these studies all demonstrated the important role of CXCR3 in cancer development. To better understand these results, we further summarized the role of CXCR3 in previous studies (25, 35). CXCR3 expression was obviously associated with immune infiltration. The overexpression of CXCR3 might decrease proportion of Th2 cells and IL-4 level, reducing M2 macrophage infiltration and increased the dendritic cell and tumor-infiltrating lymphocyte. We also found the CXCR3 had two different variants which played distinct biological functions (38, 39). The tumor-driven changes influenced the expression of the CXCR3 variants and their ligands promote cancer progression, which were worthy of further investigation.

Our research had some limitations. First, there are more than 40 immune components in the tumor microenvironment. In addition, the immunoregulatory mechanisms could not be fully explained in our study.

## Conclusions

In conclusion, our study proposed and verified three novel immune classes (the Immunity-H group, the Immunity-M group, and the Immunity-L group) of HNSCC according to 40 immune components in the tumor environment. Patients in the Immunity-H group responded to monotherapy of radiotherapy, chemotherapy or immunotherapy, and combination radiotherapy and chemotherapy or immunotherapy benefited patients in the Immunity-M group. Moreover, CXCR3 was found to play a significant role in immunoregulation, and CXCR3-targeted therapy may be an ideal candidate treatment to improve the prognosis of patients in the Immunity-L group.

## Data Availability Statement

Publicly available datasets were analyzed in this study. This data can be found here: TCGA-HNSC cohort https://genome-cancer.ucsc.edu/ GSE65858 and GSE41613 https://www.ncbi.nlm.nih.gov/.

## Ethics Statement

The study was approved by the Ethics Committee of the First Hospital of China Medical University, Shenyang, China.

## Author Contributions

PW and QQ have made substantial contributions to research design. PW, YW, and YJ and have made substantial contributions to data extraction and analysis. PW and QQ have been involved in drafting the manuscript and modification. All authors contributed to the article and approved the submitted version.

## Funding

This research was supported by the Key Research and Development Plan of Liao Ning province (grant no.2017225023), Wu Jieping Medical Foundation (320.6750.2020-05-8) and CSCO-HENGRUI Oncology Research Foundation (Y-HR2019-0326).

## Conflict of Interest

The authors declare that the research was conducted in the absence of any commercial or financial relationships that could be construed as a potential conflict of interest.
